# Association between reductions in low-density lipoprotein cholesterol with statin therapy and the risk of new-onset diabetes: a meta-analysis

**DOI:** 10.1038/srep39982

**Published:** 2017-01-10

**Authors:** Shaohua Wang, Rongrong Cai, Yang Yuan, Zac Varghese, John Moorhead, Xiong Z. Ruan

**Affiliations:** 1 Department of Endocrinology, the affiliated Zhongda Hospital of Southeast University, No. 87 DingJiaQiao Road, Nanjing, P.R. China; 2 AstraZeneca-Shenzhen University Joint Institute of Nephrology, Centre for Nephrology & Urology, Department of Physiology, Shenzhen University Health Science Center, Shenzhen, P.R. China; 3 John Moorhead Research Laboratory, Centre for Nephrology, University College London Medical School, Royal Free Campus, London, UK

## Abstract

A recent meta-analysis demonstrated that statin therapy was associated with a risk of diabetes. The present study investigated whether the relative reduction in low-density lipoprotein cholesterol (LDL-c) was a good indicator of the risk of new-onset diabetes. We searched the PubMed, Embase, Cochrane Central Register, Lilacs, Food and Drug Administration, and European Medicines Agency databases for randomized controlled trials of statins. Fourteen trials were included in the study. Eight trials with target LDL-c levels ≤100 mg/dL (2.6 mmol/L) or LDL-c reductions of at least 30% were extracted separately. The results showed that the overall risk of incident diabetes increased by 11% (OR = 1.11; 95% CI 1.03–1.20). The group with intensive LDL-c-lowering statin had an 18% increase in the likelihood of developing diabetes (OR = 1.18; 95% CI, 1.10–1.28). Furthermore, the risks of incident diabetes were 13% (OR = 1.13; 95% CI 1.01–1.26) and 29% (OR = 1.29; 95% CI 1.13–1.47) in the subgroups with 30–40% and 40–50% reductions in LDL-c, respectively, suggesting that LDL-c reduction may provide a dynamic risk assessment parameter for new-onset diabetes. In conclusion, LDL-c reduction is positively related to the risk of new-onset diabetes. When LDL-c is reduced by more than 30% during lipid-lowering therapy, blood glucose monitoring is suggested to detect incident diabetes in high-risk populations.

Many large-scale clinical trials have been performed since the Food and Drug Administration (FDA) approved the first statin to lower cholesterol levels in 1987. These clinical statin trials have shown that statins have powerful cardio protective effects in the general population^1^,^2^,^3^,^4^,^5^. Several studies^1^,^3^ have reported a low risk of coronary heart disease (CHD) with intensive low-density lipoprotein cholesterol (LDL-c)-lowering therapy. Therefore, in patients with a high risk of cardiovascular disease, the European Guidelines on cardiovascular disease prevention in clinical practice recommend a LDL-c goal of <100 mg/dL (2.6 mmol/L) or a ≥30% reduction in LDL-c^6^. However, the Justification for the Use of Statins in Prevention: an Intervention Trial Evaluating Rosuvastatin (JUPITER)^7^ study reported that some participants using rosuvastatin developed diabetes. Additionally, a meta-analysis in 2010 of 13 trials involving 91,140 individuals showed that statin therapy was associated with a 9% increased risk of new-onset type 2 diabetes mellitus (T2DM) over a 4-year period compared with that of patients randomized to the placebo or standard care groups^8^. Interestingly, no relationship was found between standard LDL-c lowering with statin therapy [trials with a target LDL-c level >100 mg/dL (2.6 mmol/L) or relative LDL-c reduction <30%] and incident diabetes compared with that of the placebo^9^. Recipients of intensive-dose statin therapy are generally at a high risk of both cardiovascular disease and incident diabetes. A further meta-analysis of five trials reported an increased risk of incident diabetes among those treated with intensive-dose statin therapy compared with the risk of those receiving moderate-dose statin therapy^10^.

However, statin dose may not necessarily be associated with incident diabetes. Doubling the statin dose was associated with only a 5–6% greater decrease in LDL-c^11^. Furthermore, approximately 20% of patients fail to respond to statins and appear to be ‘statin-resistant’. In this context, we previously demonstrated that inflammatory stress may cause statin resistance^12^. Patients with chronic kidney disease (CKD), diabetes, and other inflammatory diseases are at particular risk of statin resistance. However, some patients require a standard dose of statin to achieve the recommended LDL-c goal, which may be associated with a high risk of incident diabetes. This suggests that intensive-dose statin therapy may not necessarily be associated with target LDL-c levels or a proportionately greater reduction in LDL-c.

Although a recent meta-analysis suggested that a lower target LDL-c level with intensified statin therapy was related to a higher risk of new-onset diabetes, not all patients receiving statin treatment can reach the LDL-c target, but they all may have a risk of diabetes. This is especially true in patients with high baseline levels of LDL-c. The Deutsche Diabetes-Dialyse-Studie (4D)^13^, An Assessment of Survival and Cardiovascular Events (AURORA)^14^, and the Assessment of LEscol in Renal Transplantation (ALERT)^15^ studies suggest that specific cholesterol treatment targets do not need to be recommended as the goal of statin treatment in high-risk populations. The American College of Cardiology and American Heart Association (ACC-AHA) proposed that an assessment of both 10-year and lifetime cardiovascular disease risk should be considered when taking lipid-lowering therapy and that stroke should be included in cardiovascular disease risk estimates^16^. According to the new guidelines, neither the dose of statin nor target LDL-c are good indicators; instead “appropriate intensity” statin therapy in addition to a heart-healthy lifestyle should be applied to reduce cardiovascular risk in the major statin benefit groups. “High-intensity” statin treatment, which lowers LDL-c by at least 50%, or “moderate-intensity” statin treatment, which lowers LDL-c by approximately 30–49%, are recommended for cholesterol treatment. This suggests that LDL-c reduction, which represents both baseline and target values in patients, is arguably a more important indicator in clinical practice. However, the association between LDL-c reduction and incident diabetes during the statin lipid-lowering process is unknown.

Therefore, we investigated whether intensive statin therapy causes excess incident diabetes risk and whether LDL-c reduction, a recommended indicator of cardiovascular risk in the new ACC-AHA cardiovascular prevention guidelines^16^, is the most appropriate indicator for the assessment of risk of statin-induced diabetes during lipid-lowering therapy.

## Results

### Study selection

**Figure 1:**
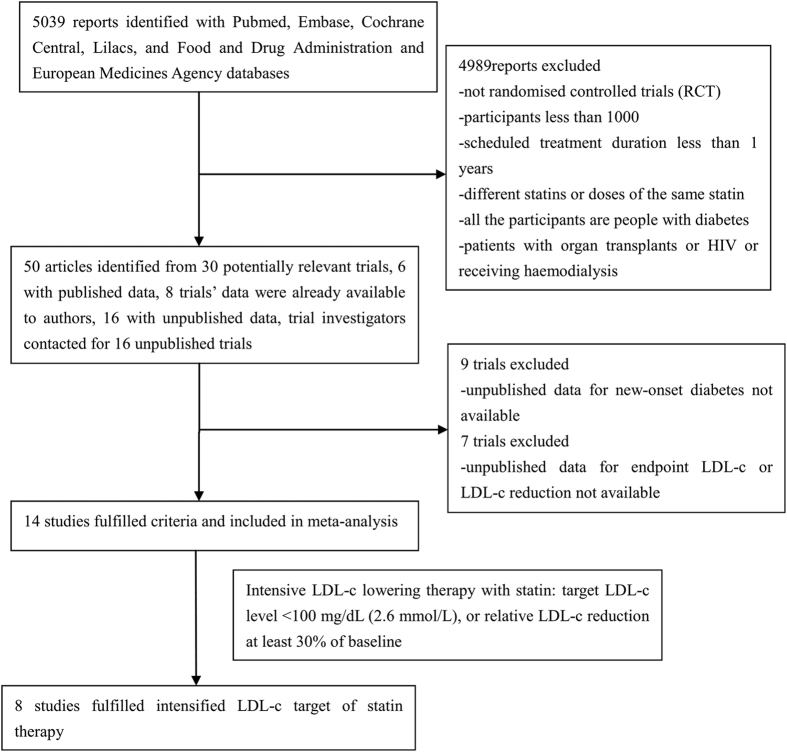
Flow diagram of literature search to identify randomized placebo-controlled and standard care-controlled statin trials.

Fourteen trials [Heart Protection Study (HPS)^17^, the Anglo-Scandinavian Cardiac Outcomes Trial—Lipid Lowering Ar (ASCOT-LLA)^18^, the Controlled Rosuvastatin Multinational Trial in Heart Failure (CORONA)^19^, JUPITER^7^, the Scandinavian Simvastatin Survival Study (4 S)^1^, the PROspective Study of Pravastatin in the Elderly at Risk (PROSPER)^20^, Gruppo Italiano per lo Studio della Sopravvivenza nell’Infarto Miocardico-Heart Failure (GISSI-HF)^21^, the Stroke Prevention by Aggressive Reduction in Cholesterol Levels (SPARCL)^22^, the West of Scotland Coronary Prevention Study (WOSCOPS)^23^, the Long-Term Intervention with Pravastatin in Ischaemic Disease (LIPID)^24^, Air Force/Texas Coronary Atherosclerosis Prevention Study (AFCAPS/TexCAPS)^5^, the Antihypertensive and Lipid-Lowering Treatment to Prevent Heart Attack Trial (ALLHAT-LLT)^25^, Members of The Management of Elevated Cholesterol in the Primary Prevention Group of Adult Japanese (MEGA)^26^, and Gruppo Italiano per lo Studio della Sopravvivenza nell’Infarto Miocardico Prevenzione (GISSI PREVENZIONE)^27^] were included in this study. Eight of the 14 trials achieved target LDL-c levels of ≤100 mg/dL (2.6 mmol/L) or intensive LDL-c reductions of at least 30% of baseline according to the guidelines for intensive LDL-c-lowering with statin therapy published in 2011^6^ (Fig. 1).

### Study characteristics

Fourteen trials with a total of 95,102 non-diabetic participants were included and were stratified according to the relative LDL-c reductions of 10–20%; >20–30%; >30–40%; and >40–50%. The baseline characteristics are presented in Table 1. According to the improved Jadad scoring method, all of the included studies had a score ≥3. In the 8 trials that met the intensive LDL-c-lowering criteria, a total of 60,287 non-diabetic participants were included, of whom 30,142 were assigned to a statin group and 30,145 to a placebo group.

### New-onset diabetes stratified by relative LDL-c reduction

**Figure 2:**
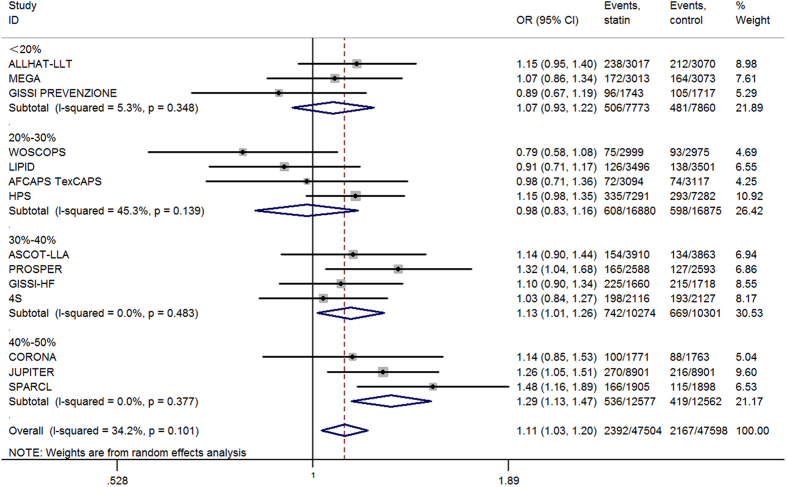
Association between different LDL-c reduction and incident diabetes.

Of the initial non-diabetic participants, 4,559 developed diabetes (Table 1). The overall risk of incident diabetes increased by 11% (OR = 1.11; 95% CI 1.03–1.20). Using the new ACC-AHA cardiovascular prevention guidelines, we stratified the 14 included trials by their relative LDL-c reduction, with categories of 10–20%; >20–30%; >30–40%; and >40–50%. The risk of diabetes was elevated by 13% (OR = 1.13; 95% CI 1.01–1.26) and 29% (OR = 1.29; 95% CI 1.13–1.47) when the relative LDL-c reductions were 30–40% and 40–50% of baseline, respectively. However, the risk of incident diabetes did not increase when relative LDL-c reductions were <20% (OR = 1.07; 95% CI 0.93–1.22) or between 20 and 30% (OR = 0.98; 95% CI 0.83–1.16) (Fig. 2). In absolute terms, over 4 years of statin therapy, one additional case of diabetes was diagnosed per 137 patients with a 30–40% relative reduction in LDL-c and one per 108 patients with a 40–50% relative reduction in LDL-c.

### Incident diabetes from trials meeting previous intensive LDL-c-lowering criteria

**Figure 3:**
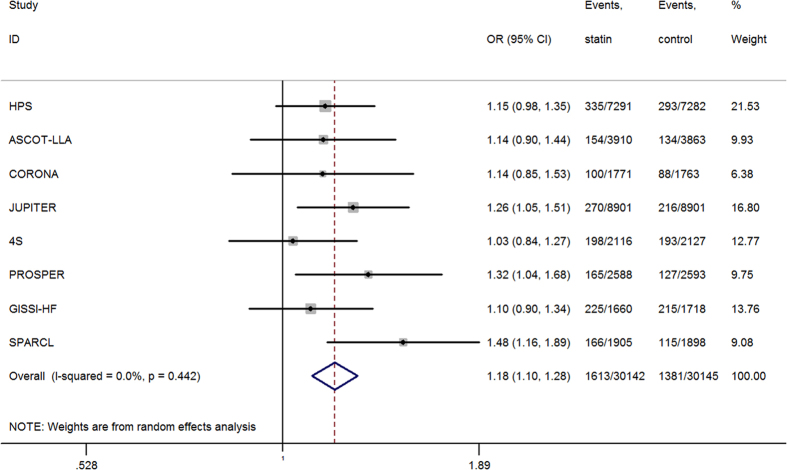
Association between intensive LDL-c lowering statin therapy and incident diabetes.

Of the 60,287 participants from 8 trials, 2,994 developed new-onset diabetes, of whom 1,613 were in the statin group and 1,381 in the placebo group. The risk of incident diabetes was higher in the intensive LDL-c-lowering statin group than that in the control group (OR = 1.18; 95% CI, 1.10–1.28; *I^2^* = 0.0%; n = 60,287) (Fig. 3). A total of 232 more cases of incident diabetes occurred in the statin-treated groups than that in the placebo-control groups, representing an 18% increase in the likelihood of participants on statin therapy developing diabetes after a mean of 4 years. For the combined study cohort, the additional 232 cases of diabetes in the statin group can also be expressed in absolute terms as one additional case of diabetes per 130 patients treated with intensive LDL-c-lowering statin therapy for 4 years. This results in 13.38 cases per 1,000 patient-years with statin treatment and in 11.45 cases per 1,000 patient-years with the placebo.

### Meta-regression analyses

As heterogeneity existed between the trials in the overall analysis, we performed meta-regression analyses of age, gender, BMI, statin dose, systolic blood pressure (BP), diastolic BP, baseline LDL-c, endpoint LDL-c, and change in LDL-c during treatment to identify other sources of the residual differences between the trials. Among these variables, the association between statin therapy and risk of new-onset diabetes was stronger in trials with patients who were older and female and those who had low baseline and endpoint LDL-c levels and high reductions in LDL-c. However, the other variables did not appear to be important factors in these analyses.

### Sensitivity analysis and publication bias

For the four stratified trial groups with different LDL-c reduction, sensitivity analyses showed that the results of the fixed-effects model were similar to those of the random-effects model regarding incident diabetes. The funnel plot and Egger’s test indicated that the publication bias of the four groups was weak when pooling data from the four stratified trial groups according to reductions in LDL-c.

**Figure 4:**
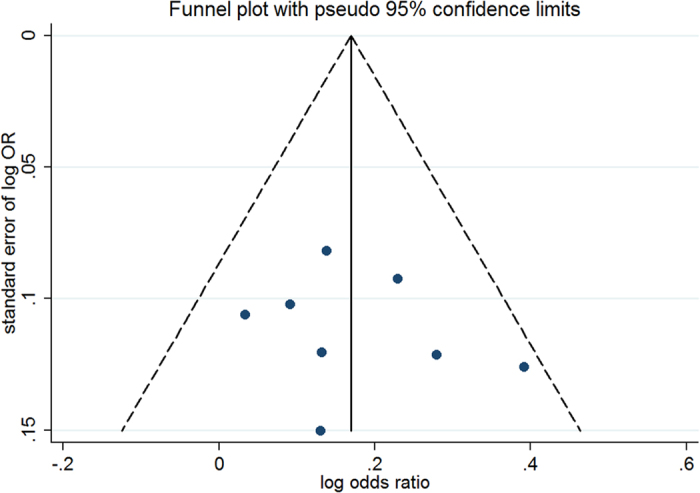
Funnel plot for trials with intensive LDL-c lowering statin therapy.

For the trials meeting intensive LDL-c-lowering statin therapy, the results of an analysis excluding studies with a high risk of bias (OR 1.20; 95% CI, 1.10–1.30; *I^2^* = 10.4%) were similar to those of the original analysis. Analyses restricted to double-blind trials and to 7 trials without HPS^17^ showed similar results as the overall analysis. The association was weakened when analysing trials without the JUPITER^7^, ROSPER^20^, and SPARCL studies^22^, possibly because of a loss of statistical power. Moreover, the combination of results of the included trials met the target LDL-c level of ≤100 mg/dL (2.6 mmol/L) or an LDL-c reduction of at least 30% of baseline in the meta-analysis, which was appropriate because the analysis of individual statins yielded overlapping CIs. Further analysis of lipophilic (OR 1.17; 95% CI, 1.06–1.29; *I^2^* = 39.0%) and hydrophilic (OR 1.20; 95% CI, 1.08–1.34; *I^2^* = 0.0%) statins showed no significant difference in terms of incident diabetes. A fixed-effects model was also used to conduct a sensitivity analysis of the intensive LDL-c-lowering statin therapy trials (OR = 1.19; 95% CI 1.10–1.28). The results of the fixed-effects model were similar to those of the random-effects model, indicating that our data had good stability. The fail-safe number in the overall analysis of the 8 trials was 53 (*P* = 0.05). The funnel plot (Fig. 4) was symmetrical, and the points were all distributed inside the 95% CI (*P* = 0.56 with Egger’s test), suggesting that the publication bias in this analysis was weak.

## Discussion

The relationship between statin therapy and incident diabetes has attracted attention because of the broader use of statins. Recently, several clinical trials examined the effect of statins on new-onset diabetes, but they showed conflicting results^7^,^23^. In the JUPITER^7^ trial, many more participants in the statin group developed diabetes than those in the placebo group. In contrast, the WOSCOPS^23^ trial showed that pravastatin may reduce the incidence of diabetes. However, an increasing number of studies have shown an enhanced risk of incident diabetes with statin therapy. Data from 285,864 men and women indicated that statin therapy was associated with a 14% increased risk of T2DM^28^. Another study conducted by Wang *et al*. also found that statin treatment was associated with an increased risk of new-onset diabetes^29^. A previous meta-analysis demonstrated that statin therapy led to a 9% increased risk of new-onset T2DM^8^, which is similar to the 11% in patients with intensive and non-intensive LDL-c-lowering statin therapy found in the present study. Our study confirms that statin therapy increases the risk of new-onset diabetes in some patients.

To investigate the factors related to statin-induced diabetes, we analysed 8 trials with intensive LDL-c-lowering statin therapy and found an 18% higher risk of incident diabetes. This finding represented a 9% higher risk of incident diabetes in patients with intensive LDL-c-lowering therapy than the risk found in a previous meta-analysis that combined intensive- and non-intensive LDL-c-lowering statin therapy. Comparing the two studies above, one case of new-onset diabetes was diagnosed in every 130 patients with intensive LDL-c-lowering therapy in our study, and one case of new-onset diabetes was diagnosed for every 255 patients in the previous study^8^. This disparity may be attributed to the differences in percentage change in the LDL-c level. In the previous study, the relative LDL-c reductions ranged from 11.5% to 50%. More than half of the included studies reported relative LDL-c reductions less than 30%. However, in our analysis of trials with intensive lowering of LDL-c, the relative LDL-c reductions ranged from 29.4% to 50%, and only 12.5% of the trials had a relative LDL-c reduction less than 30%. Therefore, it appeared that larger decreases in LDL-c accounted for the additional cases of diabetes. Other than the effects of the intensive lipid-lowering statins, the additional 9% incidence in T2DM could also be due to the extension in life, as intensive LDL-c-lowering therapy provides greater cardiovascular benefits.

Similar to our previous study^9^, in which we found that the risk of diabetes increased by 33% and 16% when intensified target LDL-c levels were ≤70 mg/dL (1.8 mmol/L) and between 70 mg/dL (1.8 mmol/L) and 100 mg/dL (2.6 mmol/L), respectively, the current study found that the risk of incident diabetes was elevated by 29% and 13% when the relative reductions in LDL-c were 40–50% and 30–40% of baseline, respectively. One plausible explanation for the similar results is that trials that achieved target LDL-c levels less than 70 mg/dL (1.8 mmol/L) were similar to trials that achieved relative LDL-c reductions of 40–50%. The same reasoning could apply to trials with target LDL-c levels between 70 mg/dL (1.8 mmol/L) and 100 mg/dL (2.6 mmol/L) and those with LDL-c reductions of 30–40%. Although our previous study suggested that a lower intensity LDL-c target level with statin therapy was associated with a higher risk of incident diabetes, the achievement of target LDL-c levels does not necessarily lead to cardiovascular protection, especially in patients with CKD, diabetes, or other inflammatory conditions^12^. Evidence suggested that chronic inflammation causes redistribution of cholesterol from the circulation to tissue compartments^30^. In this setting, circulating LDL-c is not associated with intracellular cholesterol and is inversely associated with cardiovascular risk in patients with inflammation^12^, suggesting that target LDL-c is not a good risk assessment indicator. The use of a specific LDL-c target may lead to under-treatment of certain groups or overtreatment and use of additional medications that have not been shown to add incremental or additional benefits. Although a lower LDL-c target level, which is associated with a higher risk of diabetes, not all patients receiving statin treatment can reach LDL-c targets, despite the fact that they are still at risk of diabetes. This is especially true for patients with high baseline levels of LDL-c. What’s more, not all patients who reach target LDL-c levels have a high risk of incident diabetes. Patients who reach target LDL-c levels do not necessarily have a high risk of incident diabetes if they have low LDL-c baseline levels and experience small reductions in LDL after treatment, suggesting that the target LDL-c level may not be a good indicator for the assessment of incident diabetes risk. Rather than set the LDL-c level at a particular target, the new guidelines^16^ support the application of an “appropriate intensity” therapy as the goal of statin treatment in addition to a heart-healthy lifestyle. “High-intensity” statin treatment, which lowers LDL by at least 50%, and “moderate-intensity” statin treatment, which aims to reduce LDL-c by approximately 30–49%, are recommended in “major statin benefit groups” in particular. The four major statin benefit groups include those with (1) clinical atherosclerotic cardiovascular disease (ASCVD); (2) a primary elevation of LDL-c of 190 mg/dL (4.9 mmol/L) or higher, including those with familial hypercholesterolemia; (3) diabetes, an age of 40–75 years, absence of clinical ASCVD, and LDL levels from 70–189 mg/dL (1.8–4.9 mmol/L); and (4) no clinical ASCVD or diabetes, aged 40–75 years, with an LDL of 70–189 mg/dL (1.8–4.9 mmol/L) and an estimated 10-year risk of ASCVD of at least 7.5%. With the exception of the diabetes group 3, the statin benefit groups are also closely associated with diabetes and a high risk of diabetes.

Furthermore, Dormuth showed that higher potency statin treatment was associated with a 15% higher increase in the risk of new-onset diabetes compared with lower potency statins^31^. In Dormuth’s study, higher potency statins (rosuvastatin ≥10 mg/d, atorvastatin ≥20 mg/d, and simvastatin ≥40 mg/d) were defined as those theoretically providing ≥45% reductions in LDL-c, while others were defined as lower potency statins. Another study conducted by Preiss suggested that intensive-dose statin therapy was associated with a 12% increased risk of new-onset diabetes compared with moderate-dose statin therapy^10^. In this study, the intensive-dose statins included atorvastatin at 80 mg/d and simvastatin at 40-80 mg/d. Consistent with the trials achieving intensive LDL-c-lowering in this study, both LDL-c levels in the higher potency statin group of the first study and those of the intensive-dose statin group in the second study decreased more than 30% in most trials. However, the two studies only stratified trials by dose rather than by relative LDL-c reduction. Thus, from the previous two studies, it is difficult to claim that the new-onset diabetes caused by statins is related to high reductions in LDL-c or high statin dosages. Additionally, although both studies assessed the doses of statins and their theoretical ability to reduce LDL-c, high doses of statins are not necessarily associated with an increased reduction of LDL-c or with a high risk of incident diabetes due to ‘statin resistance’. Some patients may experience ‘statin resistance’ and fail to respond to statins. Moreover, it has been reported that doubling the dose of statins only led to an approximate 5–6% greater decrease in LDL-c^11^. Lower potency statins may also result in a high risk of incident diabetes if the individuals react well to them through high reductions in LDL-c. These findings suggest that statin dose may not be a good indicator for assessing the risk of incident diabetes.

As LDL-c reduction increases in importance, more and more clinicians will use LDL-c reduction as a substitute for target LDL-c level in risk assessments. In this study, we demonstrated that LDL-c reduction was the most appropriate indicator to assess the risk of statin-induced diabetes during lipid-lowering therapy. We demonstrated that a greater reduction in baseline LDL-c resulted in an increased risk of statin-induced diabetes, especially when the LDL-c reduction was ≥30%. The risks of incident diabetes were 13% and 29% in the subgroups with 30–40% and 40–50% reductions in LDL-c, respectively.

The mechanisms underlying statin-induced diabetes remain unclear, especially the increased risk with greater reductions in LDL-c. Our study suggested that the redistribution of cholesterol from the circulation to tissue compartments may be associated with statin-induced diabetes. Ma *et al*.^32^ demonstrated that lipid redistribution from the circulation to liver can be triggered by inflammatory stress, causing fatty liver disease and a lower plasma level of LDL-c in *Apoe* knockout mice. Greater reductions in LDL-c may result in increased transfer of LDL-c to the liver and pancreatic islets. Furthermore, LDL-c accumulated in tissue compartments (such as vessels, liver and islets) can be oxidized to form oxidized LDL-c, which is a potent pro-inflammatory chemoattractant of macrophages and T lymphocytes and can induce an inflammatory cascade that compromises insulin secretion and ultimately the structural integrity of islet beta-cells^33^. This may contribute to the development of diabetes. Other than the percentage of LDL-c reduction, the increased incidence in diabetes could also be due to an increased life-span, as intensive LDL-c-lowering therapy provides greater cardiovascular benefits. More studies are needed to elucidate the mechanism of statin-induced diabetes.

There are some limitations to this meta-analysis. First, participants in the included trials were mostly Caucasian, and the applicability of the results to other ethnic groups is uncertain. Second, some unpublished data were unavailable (e.g., inflammatory markers and liver function), which could have influenced the explanation of the results. The associations between intensive LDL-c reduction and incident diabetes may be related to comorbidity, an association that requires further investigation. Third, the diagnosis of incident diabetes varied between the trials in our study. For example, in the JUPITER^7^, HPS^17^, and CORONA^19^ trials, diabetes was diagnosed based on physician report rather than on documented biochemical analyses. This may have affected the analysis of the incidence of new-onset diabetes.

In summary, previous studies have indicated that intensive LDL-c reductions provide greater cardiovascular protection in the four major statin benefit groups^16^. We demonstrated that LDL-c reduction is the most appropriate indicator for the assessment of statin-induced diabetes risk during lipid-lowering therapy. A greater reduction in baseline LDL-c resulted in an increased risk of statin-induced diabetes, especially when the percentage of LDL-c reduction was ≥30%. Blood glucose monitoring is recommended to watch out for the risk of new-onset diabetes among the four major statin benefit groups, especially when the LDL-c reduction is greater than 30% or the target LDL-c level is less than 100 mg/dL (2.6 mmol/L).

## Methods

### Literature search

This meta-analysis was performed according to the Preferred Reporting Items for Systematic reviews and Meta-Analyses (PRISMA) guidelines^34^. We searched the PubMed, Embase, Cochrane Central Register of Controlled Trials, Lilacs, Food and Drug Administration and European Medicines Agency databases from inception until May 2016 for randomized placebo and standard care-controlled endpoint trials of statins with the term “statin” and its synonyms (hydroxymethylglutaryl-coenzyme A reductase inhibitor, statins, fluvastatin, mevastatin, compactin, pravastatin, simvastatin, lovastatin, pitavastatin, rosuvastatin, cerivastatin and atorvastatin), “glucose” and “diabetes” and its synonym diabetes mellitus as keywords.

### Selection criteria

We retrieved 5,039 initial reports and excluded statin trials that were not randomized controlled trials, that compared different statins, whether different types or different doses of the same type, that were conducted in patients with diabetes, that enrolled 1,000 or fewer participants and that had a scheduled treatment duration of less than 1 year. We excluded trials conducted in patients with organ transplants or HIV or those receiving haemodialysis. Trials that lacked data on new-onset diabetes, endpoint LDL-c or reductions in LDL-c were also excluded. Two independent reviewers performed the search, and a third reviewer resolved any discrepancies. The improved Jadad score (ranging from 0 to 5) was used to evaluate the quality of the included studies. This five-point scale assesses the following components: randomization (maximum two points), double-blinding (maximum two points), and description of withdrawals or dropouts (maximum one point)^35^.

### Data sources

After application of the exclusion criteria, 30 eligible trials remained, and of these, 14 trials^1^,^5^,^7^,^17^,^18^,^19^,^20^,^21^,^22^,^23^,^24^,^25^,^26^,^27^ had published data on incident diabetes and LDL-c levels. The investigators of the remaining nine trials were contacted about obtaining unpublished data, but no responses were received. Finally, 14 trials were included. Among the 14 trials, eight of which had an LDL-c target level ≤100 mg/dL (2.6 mmol/L) or a reduction in LDL-c of at least 30% from baseline. Information about the number of non-diabetic patients at baseline and cases of incident diabetes [characteristics of trials, clinical characteristics of the patients, therapeutic intervention (type and dose of statins), change in serum lipids, and incident diabetes] were independently abstracted from the included trials.

### Statistical analysis

Based on the Cochrane guidelines, the *I^2^* statistic [with 95% confidence interval (CI)] was used to assess the statistical heterogeneity between trials. *I*^*2*^ is obtained from the formula [(Q − df)/Q] * 100%, where Q is the chi-squared statistic and df is the degrees of freedom. We calculated an overall OR with a random-effects model as the primary outcome because the random-effects model meta-analysis provides a more conservative assessment (i.e., wide CIs) of the average effect size than the fixed-effects model analysis. However, a fixed-effects model was also calculated as part of the sensitivity analysis. We used meta-regression analyses to investigate the potential sources of heterogeneity between trials. Summary odds ratios (ORs) with 95% CIs were first computed for the trials with intensive LDL-c-lowering therapy, and then, the 14 trials were stratified according to their relative reductions in LDL-c, ranging from 10–20%; >20–30%; >30–40%; and >40–50%. An OR was calculated for each LDL-c reduction range. ORs were defined as the risk of the cumulative incidence of diabetes. The absolute number of patients needed to diagnose one additional case of diabetes was calculated as (A + B)*(C + D)/(A*D − B*C), where A was the new DM patients assigned to the statin group, B was the non-diabetic patients assigned to the statin group, C was the new DM patients assigned to the control group, and D was the non-diabetic patients assigned to the control group. The cases per 1,000 patient-years in the statin and control groups were calculated as 1000*A/4*(A + B) and 1000*C/4*(C + D). We analysed the data using Stata version 11.0.

### Assessment of the risk of bias in the included studies

We performed a comprehensive search for relevant trials to minimize bias. We assessed the risk of bias in each trial (Appendix S1) by examining the risk of selection bias (random sequence generation and allocation concealment), performance and detection bias (blinding of participants, personnel and outcome assessors), attrition bias (incomplete outcome data), and reporting bias (selective reporting). Two authors assessed study bias independently, with a third author to resolve any disagreements. To assess the publication bias in trials with a target LDL-c level ≤100 mg/dL (2.6 mmol/L) or an LDL-c reduction of at least 30% according to the ACC-AHA guidelines, we used a fail-safe number and Egger’s test for a funnel plot.

### Sensitivity analysis

In the sensitivity analysis, we performed a meta-analysis by excluding studies with a high risk of bias, restricting the studies to double-blind trials, all trials other than the HPS^17^ (for the greatest weight), and trials with negative results, and assessing the effects of individual statins separately. In addition, hydrophilic and lipophilic statins were compared. Fixed-effects models were also analysed to compared with the random-effects models as part of the sensitivity analysis.

## Additional Information

**How to cite this article**: Wang, S. *et al*. Association between reductions in low-density lipoprotein cholesterol with statin therapy and the risk of new-onset diabetes: a meta-analysis. *Sci. Rep.* **7**, 39982; doi: 10.1038/srep39982 (2017).

**Publisher's note:** Springer Nature remains neutral with regard to jurisdictional claims in published maps and institutional affiliations.

## Supplementary Material

Supplementary Appendix S1

## Figures and Tables

**Table 1:** Characteristics of non-diabetic participants in 14 trials that reported incident diabetes^a^.

	Population	Intervention	Follow-up year	Mean Age (yr)^b^	Mean BMI (kg/m^2^)	Method of DM diagnosis				Jadad score
HPS	History of CVD	40 mg simvastatin or placebo	5.0	65.0	27.2	Physician reported; Medication				5
ASCOT-LLA	Hypertension CVD risk factors	10 mg atorvastatin or placebo	3.3b,^d^	63.0^b^	28.6^b^	WHO 1999 criteria				4
CORONA	Systolic heart failure	10 mg rosuvastatin or placebo	2.7^d^	73.0^b^	27.0^b^	Physician reported				5
JUPITER	No CVD	20 mg rosuvastatin or placebo	1.9^d^	66.0^b^	28.4^d^	Physician reported (medication, positive OGTT, raised random glucose with symptoms, two fasting glucose values ≥126 mg/dL (7.0 mmol/L)				5
4 S	Previous MI or angina	20–40 mg simvastatin or placebo	5.4^d^	58.6	25.9	Physician reported;medication; one fasting glucose value ≥126 mg/dL (7.0 mmol/L)				5
PROSPER	With CVD or at high risk	40 mg pravastatin or placebo	3.2	76.0	26.5	One fasting glucose value >126 mg/dL (7.0 mmol/L); medication				5
GISSI-HF	Chronic heart failure	10 mg rosuvastatin or placebo	3.9^d^	67.0	26.7	Two fasting glucose values ≥126 mg/dL (7.0 mmol/L)				5
SPARCL	With a stroke or TIA	80 mg atorvastatin or placebo	4.9	62.7^b^	27.4^b^	two fasting glucose measurements ≥126 mg/dL (7.0 mmol/l) and at least post-baseline glucose≥36 mg/dL (2.0 mmol/l) above baseline				4
WOSCOPS	No MI, raised cholesterol	40 mg pravastatin or placebo	4.8	55.2	26.0	Two fasting glucose values ≥126 mg/dL (7.0 mmol/L); medication				4
LIPID	MI or unstable angina in previous 3 years	40 mg pravastatin or placebo	6.0	62.0^d^	NA	One fasting glucose value ≥126 mg/dL (7.0 mmol/L); medication				5
AFCAPS TexCAPS	No CVD	20–40 mg lovastatin or placebo	5.2^b^	58.0^b^	27.0^b^	Physician reported; medication; one fasting glucose value ≥126 mg/dL (7.0 mmol/L)				4
ALLHAT-LLT	CHD or CHD risk factors	40 mg pravastatin or no treatment	4.8^b^	66.3	29.9	One fasting glucose value ≥126 mg/dL (7.0 mmol/L)				3
MEGA	No CVD, raised cholesterol	10–20 mg pravastatin or no treatment	5.3	58.3	23.8	Physician reported; medication; two fasting glucose values ≥126 mg/dL (7.0 mmol/L)				3
GISSI PREVENZIONE	MI within past 6 months	20 mg pravastatin or no treatment	2.0^d^	59.3	26.5	One fasting glucose value ≥126 mg/dL (7.0 mmol/L)				3
	**Sex (men%)**^b^	**Systolic BP/Diastolic BP (mm Hg)**	**Smoking (current%)**^b^	**Relative LDL-C Reduction**^c^	**Endpoint LDL-C [mg/dL(mmol/L)]**	**New DM case**	**Number assigned statin**	**Number In control Group**	**New DM assigned statin**	**New DM in control group**
HPS	78.0	143.0/81.0	15.0	29.4%	73 (1.9)	628	7291	7282	335 (4.6%)	293 (4.0%)
ASCOT-LLA	81.2	164.2/95.0^b^	32.7	34.8%^b^	89 (2.3)	288	3910	3863	154 (3.9%)	134 (3.5%)
CORONA	76.5	129.0/76.0^b^	8.6	45.1%^b^	77 (2.0)	188	1771	1763	100 (5.6%)	88 (5.0%)
JUPITER	61.8	134.0/80.0^b^	15.8	50.0%	54 (1.4)	486	8901	8901	270 (3.0%)	216(2.4%)
4 S	81.4	138.8/83.5^b^	25.6	36.7%	116 (3.0)	391	2116	2127	198 (9.4%)	193 (9.1%)
PROSPER	48.3	154.6/83.8^b^	26.8	30.7%	97 (2.5)	292	2588	2593	165 (6.4%)	127 (4.9%)
GISSI-HF	77.4	127.0/77.0^b^	14.1	34.9%	85 (2.2)	440	1660	1718	225 (13.6%)	215 (12.5%)
SPARCL	59.7	138.7/81.7^b^	19.2	63.0%	62 (1.6)	281	1905	1898	166 (8.7%)	115 (6.1%)
WOSCOPS	100.0	135.5/84.0^b^	44.0	23.7%	139 (3.6)	168	2999	2975	75 (2.5%)	93 (3.1%)
LIPID	83.2	NA	96.0	25.0%	112 (2.9)	264	3496	3501	126 (3.6%)	138 (3.9%)
AFCAPS TexCAPS	85.0	138.0/78.0^b^	12.4	26.7%	112 (2.9)	146	3094	3117	72 (2.3%)	74 (2.4%)
ALLHAT-LLT	51.2	145.0/84.0^b^	23.2	18.1%	104 (2.7)	450	3017	3070	238 (7.9%)	212 (6.9%)
MEGA	31.6	132.2/78.6^b^	20.6	17.1%	124 (3.2)	336	3013	3073	172 (5.7%)	164 (5.3%)
GISSI PREVENZIONE	86.3	NA	11.8	11.5%	124 (3.2)	201	1743	1717	96 (5.5%)	105 (6.1%)
TOTAL						4278	45521	45619	2226 (4.9%)	2052 (4.5%)

^a^BMI = body-mass index; BP = blood pressure; CVD = cardiovascular disease; CHD = coronary artery heart disease; DM = diabetes mellitus; LDL-c = low-density lipoprotein cholesterol; MI = myocardial infarction; OGTT = oral glucose tolerance test; TIA = transient ischemic attack; WHO = World Health Organization.

^b^Data from total cohort (including diabetes at baseline).

^c^Differences between the groups in the change from baseline to time point in LDL-C.

^d^Median.
