# Supplementary material for: Association between reductions in low-density lipoprotein cholesterol with statin therapy and the risk of new-onset diabetes: a meta-analysis
Source: Sci Rep. 2017 Jan 10;7:39982. doi: 10.1038/srep39982 (PMC5223121; doi:10.1038/srep39982)
Supplement: Supplementary Appendix S1 [file srep39982-s1.doc]

**Title page**

Association between reductions in low-density lipoprotein cholesterol with statin therapy and the risk of new-onset diabetes: a meta-analysis

**Authors:**

Shaohua Wang, Ph.D^1^, Rongrong Cai, Ph.D^1^, Yang Yuan, Ph.D^1^, Zac Varghese, FRCPath^3^, John Moorhead, FRCP^3^, Xiong Z. Ruan,PhD^2,3,4*^

^1^Affiliated address: Department of Endocrinology, the affiliated Zhongda Hospital of Southeast University, No. 87 DingJiaQiao Road, Nanjing, 210009, P.R. China;

^2^Affiliated address: AstraZeneca-Shenzhen University Joint Institute of Nephrology, Centre for Nephrology& Urology, Department of Physiology, Shenzhen University Health Science Center.

^3^Affiliated address: John Moorhead Research Laboratory, Centre for Nephrology, University College London Medical School, Royal Free Campus, UK

^4^Affiliated address: Centre for Lipid Research, Key Laboratory of Molecular Biology on Infectious Diseases, Ministry of Education, Chongqing Medical University, Chongqing, P.R. China

**Author email addresses:**

Shaohua Wang : gyjwsh@126.com

Rongrong Cai: [rongrong19900710@163.com](mailto:rongrong19900710@163.com)

Yang Yuan: [nn83306779@hotmail.com](mailto:nn83306779@hotmail.com)

Zac Varghese: zac0021@gmail.com

John F. Moorhead: [jmoorhead@ashworth.vianw.co.uk](mailto:jmoorhead@ashworth.vianw.co.uk)

Corresponding author: Xiong Z. Ruan

Address: AstraZeneca-Shenzhen University Joint Institute of Nephrology, Centre for Nephrology& Urology, Department of Physiology, Shenzhen University Health Science Center and John Moorhead Research Laboratory, Centre for Nephrology, University College London Medical School, Royal Free Campus, Rowland Hill Street, London NW3 2PF, UK.

Tel: +44 (0)20 7830 2190；Fax: +44 (0)20 7830 2125

E-mail:x.ruan@ucl.ac.uk

**"Risk of bias" summary: review authors’ judgements about each risk of bias item for each included** **study.**

|  | Random sequence generation  (selection bias) | Allocation concealment  (selection bias) | Blinding  (performance bias and detection bias) | Incomplete outcome data  (attrition bias) | Selective reporting (reporting bias) |
| --- | --- | --- | --- | --- | --- |
| HPS | Low risk | Low risk | Low risk | Unclear risk | High risk |
| ASCOT-LLA | Unclear risk | Unclear risk | Low risk | Unclear risk | Low risk |
| CORONA | Low risk | Low risk | Low risk | Low risk | Low risk |
| JUPITER | Low risk | Low risk | Low risk | Low risk | Low risk |
| 4S | Low risk | Low risk | Low risk | Low risk | Low risk |
| PROSPER | Low risk | Low risk | Low risk | Low risk | Low risk |
| GISSI-HF | Low risk | Low risk | Low risk | Low risk | Low risk |
| SPARCL | Unclear risk | Unclear risk | Low risk | Low risk | Low risk |
| WOSCOPS | High risk | High risk | Low risk | Low risk | Low risk |
| LIPID | Low risk | Unclear risk | Unclear risk | Low risk | Low risk |
| AFCAPS TexCAPS | High risk | Unclear risk | High risk | Low risk | Low risk |
| ALLHAT-LLT | Low risk | High risk | High risk | Low risk | Low risk |
| MEGA | Low risk | High risk | Low risk | Unclear risk | Low risk |
| GISSI PREVENZIONE | Low risk | High risk | High risk | Unclear risk | Low risk |
